# Correlation between fluoride release, surface hardness and diametral tensile strength of restorative glass ionomer cements

**DOI:** 10.4317/jced.62161

**Published:** 2024-10-01

**Authors:** Paolo Boffano, Valeria Nikolovska, Andrea Melle, Panagiotis Stathopoulos, Muhammad Ruslin

**Affiliations:** 1 Department of Health Sciences, University of Eastern Piedmont, Novara, Italy; 2 Division of Dentistry, Vercelli Hospital, Vercelli, Italy; 3 KAT, Athens, Greece; 4 Hasanuddin University, Makassar, Indonesia

## Abstract

**Background:**

A complete and thorough understanding of head and neck anatomy by dental hygienists is fundamental for performing successful dental hygiene procedures in all clinical settings. Therefore, the aim of the present study was to assess the opinion of a population of dental hygiene students about the educational methods, their perceptions of the tooth drawing module, and their opinion about the content of Anatomy curriculum in an Italian University.

**Material and Methods:**

A comprehensive survey about was developed and electronically distributed to the dental hygiene students. The questionnaire consisted of questions on the application of anatomical knowledge in clinical practice, opinions on the contents and methods of gross anatomy education, and opinions on the tooth drawing module.

**Results:**

The survey was completely answered by 63 respondents. According to most respondents, drawing exercises helped to better understand the anatomy of the teeth, to incorporate dental anatomy more effectively, and to improve their ability to visualize tooth anatomy. Most respondents reported that molars were the most difficult teeth to be drawn. The respondents gave the maximum importance to the anatomical knowledge of the mandible, the maxilla, the masticatory muscles, the temporomandibular joint, the palate, the tongue, the salivary glands, the trigeminal nerve, and the facial nerve.

**Conclusions:**

Dental drawing exercises seem to be extremely important for Dental Hygiene Bachelor Degrees and they are well appreciated by students. Appropriate educational methods of anatomy should be used to improve the attention and the learning by dental hygiene students, thus finally hopefully resulting in the improvement of their clinical skills.

** Key words:**Dental hygiene, anatomy, drawing, teeth, education.

## Introduction

Dental hygienists are dental professionals that contribute to the obtainance, prevention, and promotion of oral and dental health (1,2).

Dental hygienists in Italy perform oral health education, preventive dental treatment, periodontal assessment, and professional dental cleaning and whitening. Worldwide, as in Italy, a two to three years bachelor’s degree in dental hygiene is the most common degree for the access to the profession of dental hygienist.

An accurate and appropriate knowledge of gross anatomy is fundamental for any medical profession to perform accurate physical examinations and diagnosis and to obtain successful treatment, as well as to allow an efficacious communication among colleagues (3-7). In Italy, most of the first- year curriculum of dental hygiene degree includes both general anatomy and head and neck anatomy. The knowledge of Anatomy is acknowledged to be crucial for a safe and competent clinical practice in all health science professions, and in particular for dental hygienists.

Dental hygiene students are required to integrate their didactic knowledge of Anatomy with their clinical motor skills and show performance improvement to achieve the competencies required to perform dental hygiene procedures (8,9).

Dental hygiene is a dental profession that needs fine motor skills, hand–eye coordination, and spatial perception, so that the understanding of the anatomy of teeth is particularly fundamental.

Dental hygiene students have to learn basic shapes and predictable patterns so that they can recognize the teeth and individuate possible anomalies or pathologies. Furthermore, the anatomical complexity and variation of the teeth may make dental anatomy a difficult subject to learn for dental hygiene students.

In some Italian dental University courses, Anatomy education for dental hygienists is implemented by dental drawing and the use of appropriate technologies, such as the Anatomage Table. In particular, teeth drawing modules are frequently included in the Anatomy course curriculums to enhance students’ understanding of tooth morphology and their dexterity, so that they learn to draw the accurate morphology of teeth. Dental hygiene students are taught to perform accurate drawings of teeth, thus including the crown and root with all the featured anatomy in each aspect of a tooth.

In the literature, research on gross anatomy education for dental hygiene students is lacking (9).

A complete and thorough understanding of head and neck anatomy by dental hygienists is fundamental for performing successful dental hygiene procedures in all clinical settings.

Therefore, the aim of the present study was to assess the opinion of a population of dental hygiene students about the educational methods, their perceptions of the tooth drawing module, and their opinion about the content of Anatomy curriculum in an Italian University.

## Material and Methods

A comprehensive survey about was developed and electronically distributed to the dental hygiene students of the first (after they completed the dental anatomy course and the tooth drawing module), second, and third year from the Dental Hygiene bachelor degree at the University of Eastern Piedmont, Vercelli, Italy via Google Form.

The dental hygiene students provided informed consent after they read the content and purpose of the study on the online consent form, and their study participation was then confirmed.

The questionnaire was constructed by the authors with referral with the questionnaires constructed by Kim and Kim (9) and by Elgendy *et al*. (1). It consisted of questions on the application of anatomical knowledge in clinical practice, opinions on the contents and methods of gross anatomy education, and opinions on the tooth drawing module.

The first section of the survey was developed to collect students’ demographic data (age, gender, year of bachelor degree).

The second section of the survey was focused on the students’ perceived educational value of the drawing exercises, the students’ opinions regarding whether the drawing exercises correlated with their manual skills, and the self-rating their drawing skills.

The third section included questions regarding the students’ opinions about the usefulness of the knowledge of Anatomy in their future profession of dental hygienists. A list of anatomical structures was then constructed: respondents answered (by a five-point Likert scale) the degree to which the knowledge of each item is necessary in performing clinical dental work. The degree of utility of various educational methods of Anatomy was answered in the same manner.

Data were electronically collected through Google Forms, imported directly into Excel, and analyzed using SPSS statistical package (IBM Corp., Armonk, NY).

Descriptive statistics were used to analyze the participants’ demographics and characteristics, as well as their opinions on educational methods of gross anatomy for conducting clinical dental practice.

Institutional Review Board for the present study was not needed according to local laws.

## Results

The survey was completely answered by 63 respondents, that were included in the study. Mean age of the study population of dental hygiene students was 24.6 years (range, 19 - 45; median, 23 years; SD, 5.4 years). On the whole, 48 respondents were females, and 15 were males. Twenty-five students were attending the first year of bachelor degree of Dental Hygiene, 16 the second year, and 22 the third year.

 Table 1 resumes the answers of the respondents to the items of the second section of the survey that was focused on the students’ perceived educational value of the drawing exercises. According to most respondents, drawing exercises helped to better understand the anatomy of the teeth (87% of “agree” and “strongly agree” answers), to incorporate dental anatomy more effectively (71% of “agree” and “strongly agree” answers), and to improve their ability to visualize tooth anatomy (64% of “agree” and “strongly agree” answers). The majority of students (52%) reported that the drawing exercises made them like the Anatomy course more: this result was confirmed by the fact the 76% of respondents were “satisfied” or “very satisfied” with their experience with dental drawing exercises. The most challenging features of drawing exercises were, almost with the same reported difficulty, both drawing itself (40%) and the anxiety to be assessed (36%).

**Figure 1:**
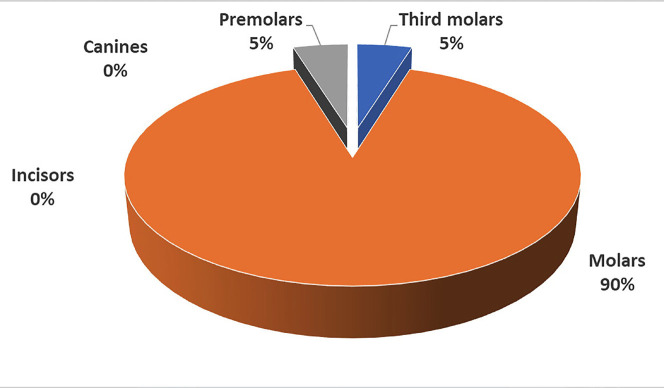
Graph showing the most difficult teeth to be drawn according to the respondents.

Most respondents (57 students; 90%) reported that molars were the most difficult teeth to be drawn, followed by third molars and premolars (both teeth with 3 respondents each) (Fig. 1).

**Figure 2:**
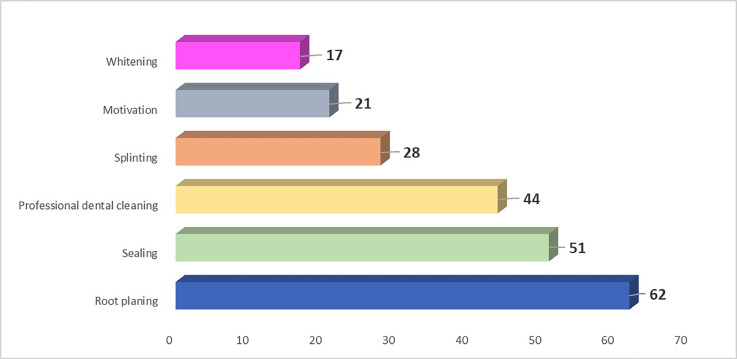
Graph showing which clinical procedures require anatomical knowledge, according to the respondents.

 Table 2 resumes the answers of the respondents regarding the utilization of anatomical knowledge in the clinical practice, while Figure 2 depicts which clinical procedures require anatomical knowledge in their opinion: only 14 students out of 63 sustained the importance of anatomical knowledge for all the six proposed dental hygiene procedures.

The opinions of the respondents about the degree to which anatomical knowledge of the following items is required to perform clinical dental work are presented in  Table 3; the respondents gave the maximum importance to the anatomical knowledge of the mandible, the maxilla, the masticatory muscles, the temporomandibular joint, the palate, the tongue, the salivary glands, the trigeminal nerve, and the facial nerve, with 70% or more of extremely positive answers (“strongly needed” or “essential”). Instead, the anatomical knowledge of hyoid bone, sphenoid bone, and temporal bone were considered not to be important to perform clinical dental work, with 35% or more of extremely negative answers (“not needed” or “barely needed”).

The opinions of dental hygiene students regarding different anatomy teaching methods are resumed in  Table 4. The respondents expressed their preference for anatomical models, 3D visualization program, and the observation of videos or photographs, giving 50% or more of extremely positive answers (“strongly needed” or “essential”). Instead, anatomy learning by dissection practice using cadavers was not considered to be a priority by most respondents, with 50% of extremely negative answers (“not needed” or “barely needed”).

## Discussion

Dental hygiene students are the principal stakeholders of the educational process of Dental Hygiene Bachelor Degrees. The perspective and the feedback of such students about the evolving educational Anatomy techniques is fundamental to improve their educational learning experience (1-4,9).

Appropriate tooth drawing may confirm that a dental hygiene student has accurately understood the external morphology of teeth. Moreover, together with the visual ability, this educational technique contributes to improve the students’ manual dexterity skills. In the literature, drawing skills have been found to be strongly associated with dental skills, with the aim of identifying the best candidates for dental schools.

In the present study, most dental hygiene students stated that dental drawing exercises helped them to better understand the anatomy of the teeth, to incorporate dental anatomy more effectively, and to improve their ability to visualize tooth anatomy. Furthermore, most students reported that the drawing exercises made them like the Anatomy course more, with 76% of respondents that were “satisfied” or “very satisfied” with their experience with this educational method.

These results seem to confirm the importance of dental drawing exercises for the education of new dental hygienists. This particular anatomical educational method seems to be greatly appreciated by students, improving their ability to understand the anatomy of teeth and together contributing to improve the students’ manual dexterity skills.

It is interesting to notice the great importance given by the respondents to the anxiety to be assessed in their ability to perform dental anatomy drawings. In Italy, this peculiar educational method is often an important subject of examination for Dental Hygiene Bachelor Degree students and it may be fundamental to allow the prosecution of their studies.

The molars have been acknowledged as the most difficult teeth to be drawn by almost all the respondents, as it was expected.

The results of  Table 2, instead, might be quite surprising as only 54% of respondents answered that they “frequently” or “very frequently” used their knowledge of anatomy in their clinical work. This result may be related to the underestimation of the role of the anatomy in the clinical work of any dental practitioner: this may explain why only 14 students out of 63 sustained the importance of anatomical knowledge for all the six proposed dental hygiene procedures. As it is shown in Figure 2, root planning seems to be the only clinical procedure in which the importance of the knowledge of anatomy is acknowledged by almost all the students. It is curious to notice that, according to most dental hygiene students, dental whitening, motivation to oral hygiene, and dental splinting would not require an accurate knowledge of anatomical concepts. For all these three procedures, dental practitioners need to use several anatomical notions in order to accurately perform a dental whitening or a dental splinting or to explain how to clean the teeth and its importance.

The results about the degree to which anatomical knowledge of the following items is required to perform clinical dental work were expected, with hyoid bone, sphenoid bone, and temporal bone as the least important to perform clinical dental work, according to dental hygiene students. Instead, the respondents gave the maximum importance to the anatomical knowledge of the mandible, the maxilla, the masticatory muscles, the temporomandibular joint, the palate, the tongue, the salivary glands, the trigeminal nerve, and the facial nerve. As observed in the previous literature (1,9), the gradual increase in the number of patients with temporomandibular disorder may have determined this answer in dental hygiene students that consider the knowledge of TMJ a fundamental requirement.

As for teaching methods of anatomy, the respondents expressed their preference for anatomical models, 3D visualization program, and the observation of videos or photographs, whereas they did not consider anatomy learning by dissection practice using cadavers to be a priority. Of course, it has to be noticed that the students included in the study commonly use the Anatomage  Table 3, Table 4 during their bachelor degree, so their answers to such questions may be influenced by their appreciation of this particular technology. Furthermore, traditionally, in Italy the use of dissection practice using cadavers has been often neglected in most medical professional degrees.

The teaching of gross anatomy to dental hygiene students should be accompanied by education methods focused on topics directly related to clinical procedures. Students should have at least a basic knowledge of the clinical field to appropriately understand the subject. Furthermore, the identification of anatomical structures on normal radiographs in addition to providing general theory may help students in strengthening their clinical competence.

## Conclusions

Dental drawing exercises seem to be extremely important for Dental Hygiene Bachelor Degrees and they are well appreciated by students. This education method of anatomy may improve the manual dexterity and the spatial understanding of anatomical information, as well as the knowledge of tooth anatomy. The importance of an accurate knowledge of anatomy for the clinical practice of any dental profession should be stressed.

Appropriate educational methods of anatomy should be used to improve the attention and the learning by dental hygiene students, thus finally hopefully resulting in the improvement of their clinical skills.

## Figures and Tables

**Table 1:** Students’ perceived educational value of drawing exercises.

Questions	Distribution (%)										
	Strongly disagree	Disagree		Slightly disagree		Slightly agree		Agree			Strongly agree
Q1: Before starting the drawing exercises, I thought I would be able to pass the exercises.	4 (6%)	8 (13%)		15 (24%)		16 (25%)		16 (25%)			4 (6%)
Q2: Participating in the drawing exercises improved my manual clinical skills.	7 (11%)	6 (9%)		12 (19%)		11 (17%)		21 (33%)			6 (9%)
Q3: The drawing exercise helped me to better understand the anatomy of the teeth.	1 (2%)	1 (2%)		2 (3%)		4 (6%)		34 (54%)			21 (33%)
Q4: The drawing exercise helped me to better understand the occlusion course.	3 (5%)	3 (5%)		11 (17%)		12 (19%)		28(44%)			6 (9%)
Q5: The drawing exercises helped me incorporate dental anatomy more effectively.	1 (2%)	2 (3%)		5 (8%)		10 (16%)		22 (35%)			23 (36%)
Q6: I developed better fine motor skills by completing the drawing exercises	8 (13%)	9 (14%)		16 (25%)		12 (19%)		11 (17%)			7 (11%)
Q7: My ability to visualize tooth anatomy details improved because of the drawing exercises.	4 (6%)	3 (5%)		2 (3%)		14 (22%)		27 (43%)			13 (21%)
Q8: The drawing exercises should be continued with future students.	3 (5%)	3 (5%)		5 (8%)		15 (24%)		16 (25%)			21 (33%)
	It made me like the course less			It had no impact				It made me like the course more			
Q9: How did the drawing exercises impact your perception of the dental anatomy course?	10 (16%)			20 (32%)				33 (52%)			
	Very poor		Poor		Fair		Good			Excellent	
Q10: How would you rate your skills on the drawing exercises?	1 (2%)		8 (13%)		23 (36%)		31 (49%)			0	
	Very dissatisfied		Dissatisfied		Satisfied				Very satisfied		
Q11: Overall, how satisfied or dissatisfied are you with your experience with the drawing exercises?	2 (3%)		13 (21%)		42(67%)				6 (9%)		
	Drawing	Understanding of Anatomy			Anxiety to be evaluated				Other		
Q12 In your opinion, what is the most challenging and difficult part of teeth drawing exercises?	25 (40%)	10 (16%)			23 (36%)				5 (8%)		

**Table 2:** Distribution of answers regarding the utilization of anatomical knowledge in the clinical practice.

Questions	Distribution (%)				
Not used	Barely used	Moderate use	Frequent use	Very frequent use
Do you use your knowledge of anatomy in your clinical work?	1 (2%)	1 (2%)	27 (43%)	25 (40%)	9 (14%)

**Table 3:** Distribution of answers about the degree to which anatomical knowledge of the following items is required to perform clinical dental work, according to the respondents.

	Distribution (%)				
	Not needed	Barely needed	Moderately needed	Strongly needed	Essential
Mandible	0	5 (8%)	16 (25%)	20 (32%)	22 (35%)
Maxilla	0	5 (8%)	14 (22%)	22 (35%)	22 (35%)
Palatine bone	10 (16%)	3 (5%)	20 (32%)	23 (36%)	7 (11%)
Hyoid bone	13 (21%)	13 (21%)	26 (41%)	9 (14%)	2 (3%)
Sphenoid bone	14 (22%)	16 (25%)	22 (35%)	10 (16%)	1 (2%)
Temporal bone	9 (14%)	14 (22%)	19 (30%)	15 (24%)	6 (9%)
Facial muscles	3 (5%)	3 (5%)	21 (33%)	26 (41%)	10 (16%)
Masticatory muscles	3 (5%)	1 (2%)	15 (24%)	31 (49%)	13 (21%)
Neck muscles	7 (11%)	9 (14%)	29 (46%)	15 (24%)	3 (5%)
Temporomandibular joint	0	3 (5%)	9 (14%)	23 (36%)	28 (44%)
Palate	5 (8%)	3 (5%)	11 (17%)	18 (29%)	26 (41%)
Tongue	0	4 (6%)	10 (16%)	20 (32%)	29 (46%)
Salivary glands	0	5 (8%)	13 (21%)	22 (35%)	23 (36%)
Artery	6 (9%)	13 (21%)	18 (29%)	20 (32%)	6 (9%)
Vein	7 (11%)	13 (11%)	18 (29%)	20 (32%)	5 (8%)
Lymph	5 (8%)	14 (22%)	27 (43%)	15 (24%)	2 (3%)
Trigeminal nerve	2 (3%)	6 (9%)	9 (14%)	19 (30%)	27 (43%)
Facial nerve	4 (6%)	5 (8%)	12 (19%)	20 (32%)	22 (35%)
Vagus nerve	7 (11%)	7 (11%)	16 (25%)	23 (36%)	10 (16%)
Glossopharyngeal nerve	7 (11%)	4 (6%)	17 (27%)	23 (36%)	12 (19%)
Hypoglossal nerve	6 (9%)	5 (8%)	16 (25%)	24 (38%)	12 (19%)

**Table 4:** Distribution of answers about the opinion of the respondents on different anatomy teaching methods.

Educational methods of gross anatomy	Distribution (%)				
	Not needed	Barely needed	Moderately needed	Strongly needed	Essential
Dental hygienists must learn not only about the head and neck, but also whole-body anatomy.	7 (11%)	14 (22%)	28 (44%)	10 (16%)	4 (6%)
Team-based learning activities (quiz, games, etc.) would be useful.	9 (14%)	10 (16%)	25 (40%)	16 (25%)	3 (5%)
Gross anatomy and other clinical subjects (clinical dental sciences, oral radiology, etc.) need integrated education.	6 (9%)	8 (13%)	26 (41%)	13 (21%)	10 (16%)
Dissection practice using cadavers is required.	18 (29%)	13 (21%)	12 (19%)	10 (16%)	10 (16%)
Practice through observations of anatomical models is necessary.	1 (2%)	5 (8%)	20 (32%)	19 (30%)	18 (29%)
Anatomy practice using a 3D visualization program is necessary.	1 (2%)	4 (6%)	11 (17%)	18 (29%)	29 (46%)
Anatomy practice through observations of videos or photographs (X-rays, CT, MRI, etc.) is necessary.	1 (2%)	4 (6%)	9 (14%)	25 (40%)	24 (38%)

## Data Availability

The datasets used and/or analyzed during the current study are available from the corresponding author.
